# The BIDIAP index: a clinical, analytical and ultrasonographic score for the diagnosis of acute appendicitis in children

**DOI:** 10.1007/s00383-023-05463-5

**Published:** 2023-04-10

**Authors:** Javier Arredondo Montero, Carlos Bardají Pascual, Giuseppa Antona, Raquel Ros Briones, Natalia López-Andrés, Nerea Martín-Calvo

**Affiliations:** 1 Pediatric Surgery Department, Hospital Universitario de Navarra, Calle Irunlarrea 3, 31008 Pamplona, Navarra Spain; 2 Department of Preventive Medicine and Public Health, School of Medicine, University of Navarra, Pamplona, Navarra, Spain; 3 Cardiovascular Translational Research, NavarraBiomed (Miguel Servet Foundation), Hospital Universitario de Navarra, Universidad Pública de Navarra (UPNA), IdiSNA, Pamplona, Navarra, Spain; 4 IdiSNA, Instituto de Investigación Sanitaria de Navarra, Pamplona, Navarra, Spain; 5 CIBER de Fisiopatología de la Obesidad y la Nutrición, Instituto de Salud Carlos III, Madrid, Spain

**Keywords:** BIDIAP index, Systemic immune-inflammation index, Appendicular caliber, Peritoneal irritation, Pediatric acute appendicitis, Collinearity, Score

## Abstract

**Background:**

Pediatric acute appendicitis (PAA) continues to be a diagnostic challenge today. The diagnostic performance of classical indices is only moderate, especially in pediatric population. This study aimed to define a clinical, radiological and analytical index for the diagnosis of PAA.

**Materials and methods:**

This prospective study included 151 patients divided into two groups: (1) 53 patients with non-surgical abdominal pain (NSAP) and (2) 98 patients with a confirmed PAA. Sociodemographic and clinical characteristics were compared between groups using the Mann–Whitney *U* test and the Fisher exact test. To identify the predictors of PAA, we performed a multivariable logistic regression using a *forward stepwise* analysis and we assigned multiples of integer values to the selected variables. The diagnostic performance of the index was assessed by calculating the area under the receiver operating characteristic curve. Intra-cohort calibration was assessed with the Hosmer–Lemeshow test.

**Results:**

We developed the BIDIAP index (BIomarkers for the DIagnosis of Appendicitis in Pediatrics), which included three variables that independently predicted higher odds of PAA: appendiceal caliber (≥ 6.9 mm), systemic immune-inflammation index (≥ 890) and peritoneal irritation, which scored 4, 3 and 2 points, respectively. Mean (SD) score of the participants was 2.38 (2.06) in group 1 and 7.89 (1.50) in group 2. The area under the ROC was 0.97 (95% CI 0.95–0.99). The cut-off point was established at 4 points, resulting in a sensitivity of 98.98% and a specificity of 77.78%.

**Conclusions:**

The BIDIAP index has an exceptional diagnostic performance in PAA. The importance of these results lies in its novelty and in the simplicity of the index. Although external validation will be necessary, initial results look promising.

**Supplementary Information:**

The online version contains supplementary material available at 10.1007/s00383-023-05463-5.

## Introduction

Pediatric acute appendicitis (PAA) continues to be a major diagnostic challenge nowadays. The important consequences in terms of morbidity, mortality and health-care costs attributable to misdiagnosis make this a public health problem that requires urgent attention [1].

In recent decades, multiple biomarkers have been explored as potential diagnostic tools in the context of PAA [2–5]. Although some biomarker demonstrated acceptable diagnostic yields, none showed sufficient discriminatory capacity to be considered as a unique test in the diagnosis of PAA [6, 7]. Besides, it must be noted that many of those markers are used with research purposes and that their implementation in clinical practice is not feasible because of either economic or processing time issues.

Previous studies analyzed the validity of ratios derived from the basic blood count as diagnostic tools, including the neutrophil-to-lymphocyte ratio, the platelet-to-lymphocyte ratio and the monocyte-to-lymphocyte ratio [8, 9]. Those ratios had important advantages, such as not requiring additional economic or human resources and being available from the outset for assessment. Although the neutrophil-to-lymphocyte ratio showed a good diagnostic performance, it also cannot be considered in isolation for the diagnosis of PAA.

The systemic immune-inflammation index (SII) is a novel ratio that has been proposed as a diagnostic and prognostic biomarker in different clinical situations, as neoplastic processes or autoimmune diseases [10, 11]. This ratio, which combines the absolute values of neutrophils, lymphocytes and platelets, reliably reflects the degree of systemic inflammation/immune activation. Neutrophilia is a marker of acute stress and is closely related to bacterial infections which, when pronounced, may be accompanied by lymphopenia. In addition, in systemic inflammatory conditions, platelets can act as an acute phase reactant. Given the intrinsic characteristics of PAA, we hypothesize that this ratio may be more valid than the neutrophil–to-lymphocyte ratio for the diagnosis of this pathology. To the best of our knowledge, the diagnostic performance of SII has not been assessed in PAA to date.

On the other hand, the use of specific scores such as the Alvarado score and the pediatric appendicitis score (PAS) for the PAA diagnosis is widespread. Although the Alvarado score is not specific for children, it has demonstrated adequate diagnostic performance in this population [12].The PAS, a modified version of the former for the pediatric population, has also shown good diagnostic yield, but is far from being perfect [13].

The aim of this study was to evaluate the diagnostic performance of a series of clinical, analytical and ultrasound parameters to combine them into a simple and easy-to-apply index to improve the diagnosis of PAA.

## Materials and methods

### Study design

BIDIAP (BIomarkers for the DIagnosis of Appendicitis in Pediatrics) is a prospective non-randomized observational study [14, 15]. Participants were recruited in the Emergency Department and in the Pediatric Department of our center (a tertiary-level pediatric hospital) when the personnel conducting the investigation were present. The recruitment period extended from February to December 2021. Inclusion and exclusion criteria are listed in Supplementary file 1.

Two groups of pediatric patients were included in this study: (1) patients with non-surgical abdominal pain (NSAP) (patients who were initially evaluated with a clinical suspicion of acute abdomen and in whom the presence of urgent abdominal surgical pathology was excluded) and (2) patients with histopathological confirmed diagnosis of PAA.

Peritoneal irritation was defined as the presence of Blumberg’s sign (rebound tenderness in the right iliac fossa), assessed by the physician who enrolled the patient in the study. Sociodemographic, clinical, analytical, surgical, radiological and histological variables of all patients were extracted from participants’ clinical records by the principal investigator (JAM).

All patients in group 1 were contacted 2 weeks after their inclusion in the study to ensure that they had not been diagnosed with PAA in that period. All patients in group 2 were followed up on an outpatient basis for 1 month after the intervention.

### Sample collection

A venous blood sample was obtained from each patient in an EDTA tube (3.5 mL). In all patients, it was obtained at the time of inclusion in the study during their stay in the Emergency Department. Serum samples were processed by laboratory personnel blinded to patient’s group.

### Calculation of systemic immune-inflammation index (SII)

The absolute neutrophil count (ANC) was defined as the total number of neutrophils in the complete blood count (CBC). The absolute platelet count (APC) was defined as the total number of platelets in the CBC. The absolute lymphocyte count (ALC) was defined as the total number of lymphocytes in the CBC. 

The SII was calculated as follows [10]: (ANC × APC)/ALC. To calculate the best cut-off value for SII (NSAP vs PAA) the distance on the ROC curve was calculated as the square root of [(1 − sensitivity)^2^ + (1 − specificity)^2^] and that with the shortest distance (lowest value) was considered the optimal cut-off.

### Radiological determinations

The appendiceal caliber (maximum transverse diameter), the presence of ultrasound appendicolith and the presence of ultrasound mesenteric lymphadenitis were evaluated. We did not consider other variables (such as appendicular parietal destratification or appendicular Doppler flow) because they are more operator dependent and, therefore, less useful as part of an index.

In relation to mesenteric lymphadenitis, we consider ultrasound positivity the presence of at least one lymph node greater than 1 cm of maximum axis.

Regarding, appendiceal caliber, all measurements were performed on ultrasonographic studies by the radiologist on duty. To calculate the best cut-off value for appendiceal caliber (NSAP vs PAA), the distance on the ROC curve was calculated as the square root of [(1 − sensitivity)^2^ + (1 − specificity)^2^] and that with the shortest distance (lowest value) was considered the optimal cut-off.

### Statistical analysis

For descriptive purposes, we used means and standard deviations or medians and interquartile ranges (IQR) for quantitative variables and proportions for categorical ones. Kolmogorov–Smirnov test was used to assess the normality of quantitative variables. Sociodemographic and clinical variables were compared between groups using the Fisher exact test and the Mann–Whitney *U* test.

To identify the independent predictors of PAA, a multivariable logistic regression was performed using a *forward stepwise *analysis with *p* for removal < 0.05. Continuous variables were previously dichotomized based on the best diagnostic performance cut-off to distinguish between PAA and NSAP and entered the model in increasing order of *p* value obtained in the univariate analyses. This analysis eliminated collinearity between variables to create a parsimonious model. Multiples of integer values were assigned to the variables of the index according to the beta coefficients obtained in the analysis. We assessed the discriminatory capacity of the BIDIAP index by calculating the area under the receiver operating characteristic curves (ROC). For each cut-off value the distance on the ROC curve was calculated as the square root of [(1 − sensitivity)^2^ + (1 − specificity)^2^] and that with the shortest distance (lowest value) was considered the optimal cut-off. Lastly, we performed a calibration of the BIDIAP index in our cohort using the Hosmer–Lemeshow test.

Statistical significance was settled in a *p* value < 0.05. Statistical analyses were performed with STATA 17.0 (Stata Corp LCC).

### Research ethics board committee

This study was approved by our center's clinical research ethics committee on December 18, 2020, under code PI_2020/112. The ethical principles of the Declaration of Helsinki were applied for the conduct of this research study. The parents or legal representatives of all participants signed an informed consent form prior to their inclusion in the study.

## Results

### Demographic and clinical characteristics

Among the 151 patients recruited, 17 (11%) in the NSAP group were excluded due of missing information in the appendiceal caliber. Therefore, the final sample consisted of 134 patients, divided into two groups: (1) patients with non-surgical abdominal pain in whom the diagnosis of PAA was excluded (*n* = 36) and (2) patients with a confirmed diagnosis of PAA (*n* = 98). Participants’ sociodemographic and clinical characteristics by group are shown in Table 1. Statistically significant differences were found in age (*p* = 0.13), sex (*p* = 0.07) and number of emetic episodes (*p* < 0.0001). No significant differences were observed between included and excluded children in sociodemographic and clinical variables (data not shown). None of the patients in the NSAP group developed PAA.

**Fig. 1:**
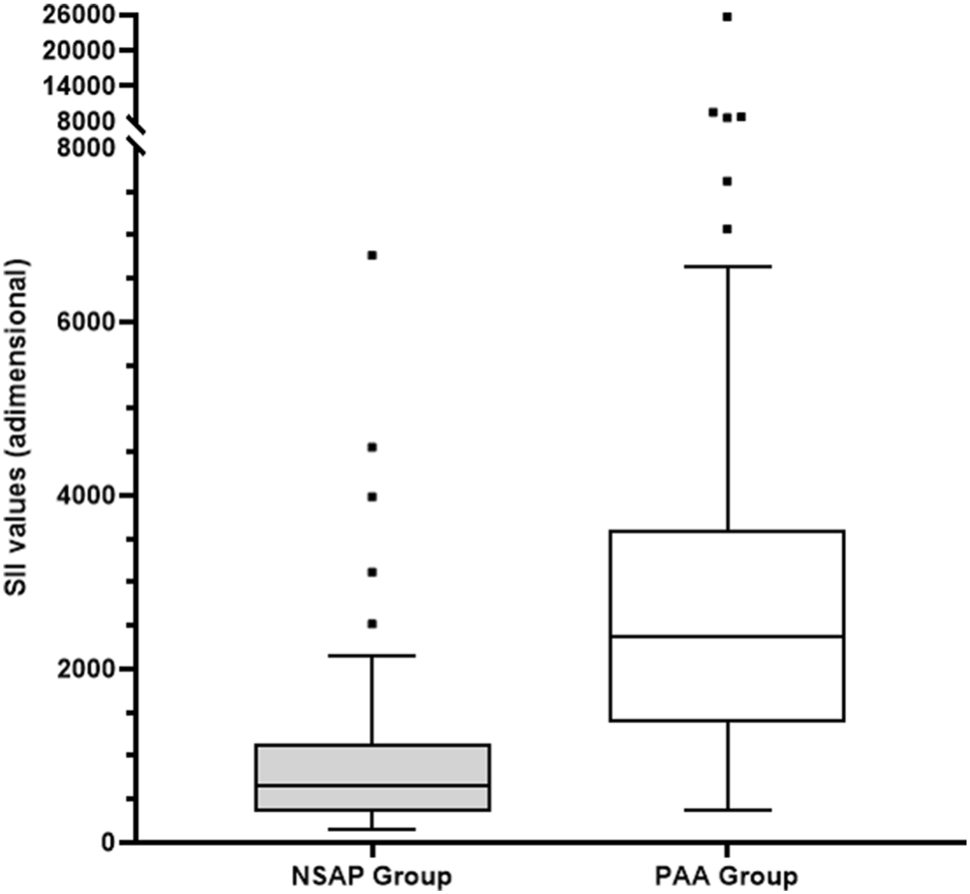
Algorithmic box plot representation of SII values in the two study groups

**Fig. 2:**
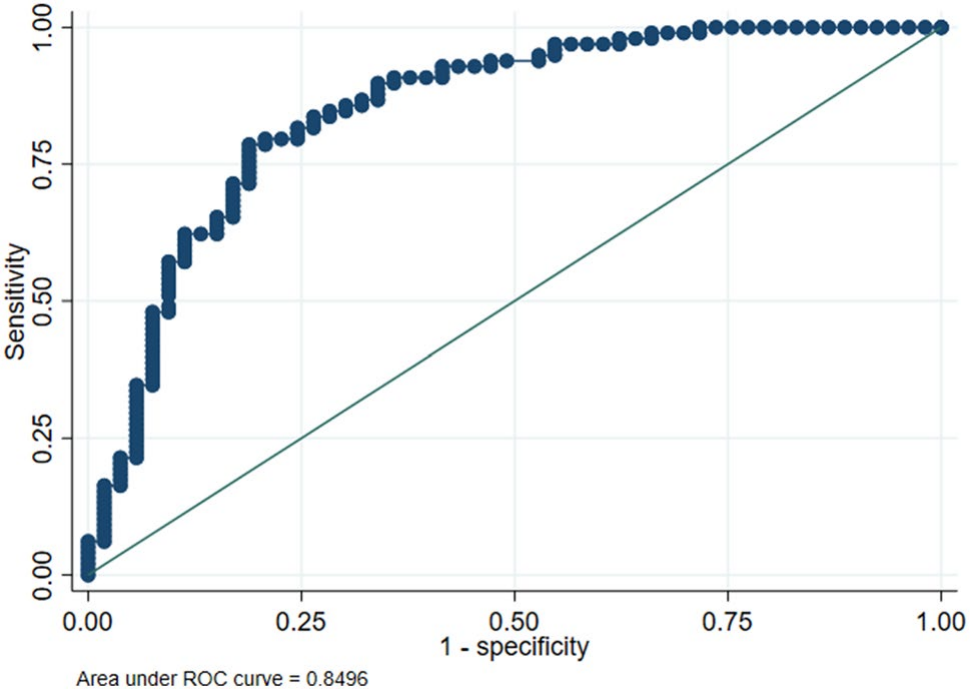
ROC curve for SII (group 1 vs 2)

Median (interquartile range) serum SII values were 696.34 (355.67–1350.38) in group 1 and 2381.85 (1409.14–3497.33) in group 2 (*p* < 0.0001). The graphical representation of SII by groups is shown in Fig. 1. A logarithmic scale was used because of the wide analytical range obtained in the determinations. The AUC for SII was 0.85 (95% CI 0.78–0.92) (*p* < 0.0001). The cutoff with the best percentage of correctly classified (81%) observations corresponded to 890, resulting in a sensitivity of 89.80% and a specificity of 66% (positive likelihood ratio: 2.64). The graphical representation of the ROC curve for SII is shown in Fig. 2.

**Table 1:** Clinical and sociodemographic characteristics of the participants of the study

Clinical and sociodemographic variables	Group 1 (NSAP) (*n* = 36)	Group 2 (PAA) (*n* = 98)	*p* value
Age (years)	10.65 (2.55)	9.69 (3.08)	0.13
Sex (male/female) (%)	17/19 (47.22%)	64/34 (65.30%)	0.07
Hours of pain evolution	29.97 (21.76)	27.08 (19.67)	0.50
Fever > 37.8 °C at home (yes/no) (%)	14/22 (38.88%)	33/65 (33.67%)	0.68
Number of diarrheal stools	0.33 (1.04)	0.63 (2.40)	0.63
Urinary symptoms (yes/no) (%)	5/31 (13.88%)	22/76 (22.44%)	0.34
Number of emetic episodes	0.42 (1.29)	2.45 (2.45)	< 0.0001
Hyporexia (yes/no) (%)	26/10 (72.22%)	79/19 (80.61%)	0.35
Leucocytes (1 × 10^9^/mL)^a^	9.65 (7.7–11.85)	16.1 (13–18.8)	< 0.0001
Neutrophils (1 × 10^9^/mL)^a^	6.15 (4.1–7.95)	13.2 (9.5–16.2)	< 0.0001
Platelets (1 × 10^9^/mL)^a^	253 (212–296)	280.5 (249–323.5)	0.008
SII index^a^	696.34 (355.67–1350.38)	2381.85 (1409.14–3597.33)	< 0.0001

Numbers are mean (standard deviation) or numbers (percentage)

^a^Median, interquartile range

The odds ratio (OR) and 95% confidence interval (CI), *p* value and the pseudo *R*^*2*^ value for PAA associated with each independent predictor are shown in Table 2. The multivariable model using a forward stepwise analysis showed that the variables that independently predicted the odds of appendicitis were: appendiceal caliber ≥ 6.9 mm, SII ≥ 890 and the presence of peritoneal irritation. The beta coefficients obtained for each of the predictors were 5.42 (appendicular caliber ≥ 6.9 mm), 3.07 (SII ≥ 890) and 2.38 (presence of peritoneal irritation). Those coefficients were divided by 2.38 (minimum common divisor) and multiplied by 2, resulting in the following equation:

$${\text{BIDIAP}}\;{\text{index}} = \, 4\,\left( {{\text{appendicular caliber }} \ge \, 6.9mm} \right) + 3\,\left( {{\text{SII }} \ge \, 890} \right) + 2\,\left( {\text{presence of peritoneal irritation}} \right).$$

**Table 2:** Potential variables that were assessed for inclusion in the score, ordered by statistical significance and pseudoR2 value

Variables	OR (95% IC)	*p* value	pseudoR2 value
Appendicular caliber ≥ 6.9 mm	115.32 (31.29–425.06)	< 0.0001	0.5607
SII index ≥ 890	17.11 (7.19–40.70)	< 0.0001	0.2629
Neutrophil-to-lymphocyte ratio index ≥ 3	14.83 (5.96–36.88)	< 0.0001	0.2187
Presence of ultrasound appendicolith	11.85 (1.51–93.1)	0.0013	0.0710
Vomiting	11.08 (4.68–26.20)	< 0.0001	0.1991
Serum interleukin-6 ≥ 19.5 pg/mL	8.76 (3.60–21.34)	< 0.0001	0.1530
Platelet to lymphocyte ratio ≥ 105	7.22 (3.33–15.64)	< 0.0001	0.1407
C-reactive protein ≥ 3 mg/dL	6.26 (2.96–13.24)	< 0.0001	0.1273
Serum pentraxin-3 ≥ 7.3 pg/mL	5.01 (2.40–10.48)	< 0.0001	0.1039
Abdominal guarding	3.45 (1.72–6.94)	0.0004	0.0642
Peritoneal irritation	3.35 (1.67–6.74)	0.0005	0.0610
No ultrasound adenopathies	2.22 (1.06–4.76)	0.034	0.0230
Hyporexia	1.96 (0.91–4.21)	0.084	0.0152
Temperature in the emergency room ≥ 37.1 °C	1.41(0.88–2.27)	0.14	0.0113
Fever at home	1.29 (0.62–2.67)	0.50	0.0024
Presence of diarrhea	1.31 (0.48–3.67)	0.59	0.0015
Right iliac fossa pain	1.06 (0.42–2.73)	0.89	0.0001
≥ 12 h from symptom onset	1.03 (0.44–2.44)	0.94	0.0001

**Fig. 3:**
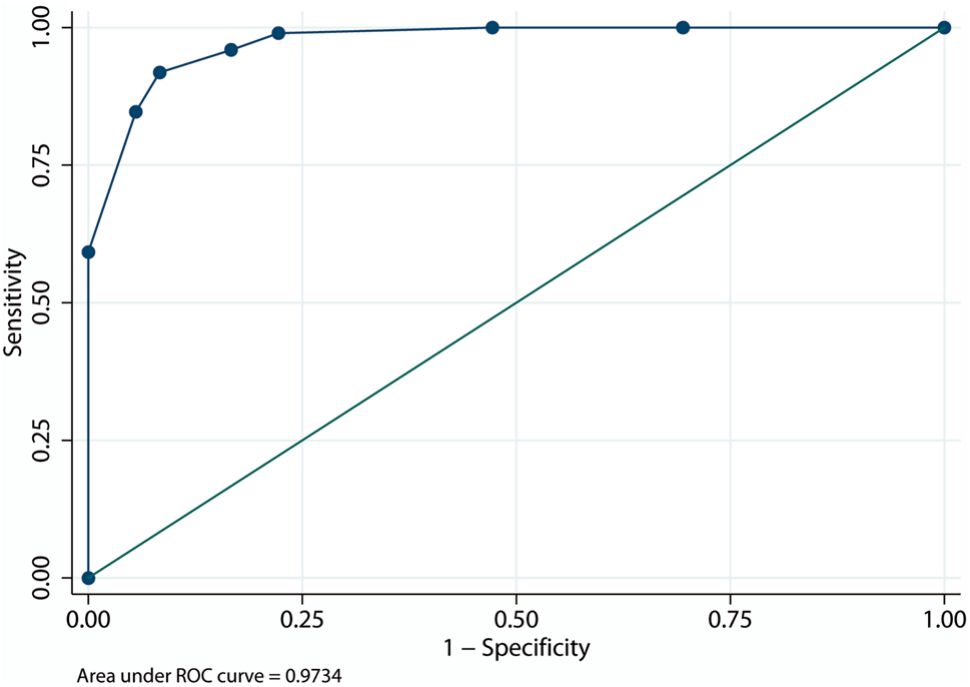
ROC curve for BIDIAP score (group 1 vs 2)

Table 3 shows the components of the BIDIAP index and their scoring weights. Mean (SD) score in the BIDIAP index was 2.38 (2.06) in the group of children with NASP and 7.89 (1.50) in the PAA group (*p* < 0.0001). The area under the curve (AUC) for the BIDIAP index was 0.97 (95% CI 0.95–0.99) (*p* < 0.001) (Fig. 3). The cut-off value with the shortest distance on the ROC curve was 4, with a sensitivity of 98.98% and a specificity of 77.78%. According to that cut-off, the diagnostic of PAA could be established if one of the following two conditions were met: either the appendiceal caliber is ≥ 6.9 mm or SII is > 890 and peritoneal irritation is present.

**Table 3:** Proposed BIDIAP index

	BIDIAP score	No	Yes
(A)	Appendicular caliber ≥ 6.9 mm	0	4
(B)	SII index ≥ 890	0	3
(C)	Presence of peritoneal irritation	0	2

The maximum value obtainable is 7 points

If the answer to (A) or (B + C) is yes, the diagnosis of PAA is confirmed

Table 4 shows alternative cut-off points in the BIDIAP index with their respective diagnostic performance in terms of sensitivity, specificity and positive likelihood ratio.

**Fig. 4:**
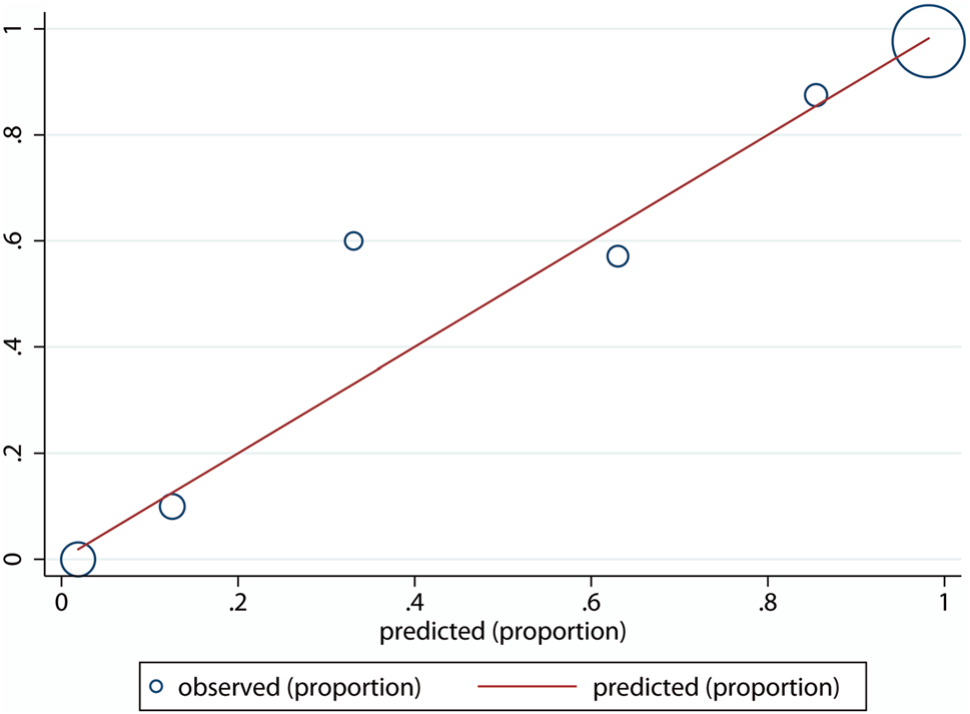
Calibration model for the SCORE (Hosmer–Lemeshow test)

The calibration test showed that the BIDIAP index fitted excellently in our sample (*p* = 0.82 in the Hosmer–Lemeshow test), as it is represented in Fig. 4.

**Table 4:** Alternative BIDIAP score cut-off values for the discrimination between NSAP and PAA. Diagnostic performance is presented in terms of sensitivity, specificity, and positive likelihood ratio

BIDIAP score cutoff value	Positive likelihood ratio	Correctly classified (%)	Sensitivity (%)	Specificity (%)
2	1.44	81.34	100	30.56
4	4.45	93.28	98.98	77.78
6	11.02	91.79	91.84	91.67
7	15.24	87.31	84.69	94.44

## Discussion

In this prospective study of 134 patients, we performed a thorough statistical analysis guided by biological plausibility criteria to identify independent predictors of PAA and to develop an easy-to-use index that showed an exceptional diagnostic performance in this pathology. The BIDIAP index includes easily accessible variables (commonly assessed in Pediatric Emergency departments), including SII, a ratio calculated from hemogram parameters whose diagnostic performance in PAA had not been previously evaluated.

To date, multiple scores have been evaluated as potential diagnostic tools in the context of PAA. The Alvarado score, initially designed for adult populations and subsequently extrapolated to the pediatric population, has shown moderate performance in the diagnosis of PAA [12]. Similar findings have been reported for PAS, a score designed specifically for the pediatric population. [16]. More recently, the appendicitis inflammatory response score (AIR score) and the pediatric appendicitis risk calculator (pARC) demonstrated diagnostic superiority over the PAS score and the Alvarado score, although clinical experience was limited [17]. The BIDIAP index has two interesting advantages that make it a better predictor of PAA than previous indices: (1) a higher diagnostic yield and (2) a remarkable simplicity, as it is composed of only three parameters and does not require specific laboratory determinations.

SII has been previously analyzed as a prognostic tool in multiple pathologies such as advanced non-small cell lung cancer [18] and extrahepatic cholangiocarcinoma [19]. In relation to its usefulness as a diagnostic tool, its application is limited, having been documented in contexts such as subacute thyroiditis [20]. To our knowledge, this is the first study assessing the diagnostic yield of the SII in PAA. In our sample, the discriminatory capacity of SII (AUC = 0.85; 95% CI 0.78–0.92) was similar to that of the neutrophil-to-lymphocyte ratio (AUC = 0.83; 95% CI 0.75–0.90), but SII showed higher OR for PAA and higher pseudo *R*^*2*^. Although more evidence is needed, our findings suggest that the SII might emerge as one of the best biomarkers for the diagnosis of PAA in clinical practice.

Our results agree with previous studies that reported that the appendiceal caliber was a strong predictor of PAA [21].

In our sample, the diagnostic performance of the appendiceal caliber alone was excellent (AUC 0.90; 95% CI 0.84–0.97). Indeed, the BIDIAP score showed that PAA diagnosis could be established even if only that condition was met (appendiceal caliber ≥ 6.9 mm). Our index adds to the previous evidence, because the diagnosis of PAA can also be conclusive when that condition is not reported as long as the other two conditions are. The ultrasound-guided visualization of the appendix in children is very technician dependent and sometimes the caliber of the appendix can be very difficult to measure [22]. Although the literature regarding the proportion of appendiceal visualization in NSAP and PAA groups is scarce, in our clinical experience it is more likely that non-visualization corresponds to NSAP cases (with a smaller appendicular caliber), since in PAA cases the appendix is usually enlarged and presents locoregional inflammatory changes that facilitate its identification. However, it is worth mentioning that recent working groups, through the implementation of specific reporting templates and education sessions, have managed to improve ultrasonographic appendix identification [23].

Regarding physical exploration, the most important clinical features assessed for the diagnosis of PAA are peritoneal irritation, defined as a positive Blumberg's sign and abdominal guarding. In our sample, peritoneal irritation (OR = 3.35; 95% CI 1.67–6.73) showed a similar association with PAA as abdominal guarding (OR = 3.45; 95% CI 1.72–6.94). Accounting for appendiceal caliber and SII, abdominal guarding was not significantly associated with the odds of PAA. This result may indicate that peritoneal irritation is usually easier to detect than abdominal guarding, especially in patients with high body mass index. In this study, participants were recruited when a member of the research team was present and physical examination was performed by the same team member following a standard procedure. Therefore, we acknowledge that, although there might be some inter-observer variability, it should be small.

Our study has several strengths including its prospective design, its large sample size and the thorough statistical analyses. The use of the statistical model presented here makes it possible to correct collinearity to a large extent, which in our opinion is one of the main problems of the diagnostic scores published to date for diagnosing PAA.

We believe that studies assessing the diagnostic performance of different biomarkers in PAA would benefit from more complex statistical analyses, such as the ones presented in this study, which allow the control of confounding and the calculation of the strength of the associations and the robustness of the results.

Despite our findings, we must acknowledge some limitations.

First, we used a convenience sampling, which is susceptible of a selection bias. Besides, 17 patients from the NSAP group (11%) were excluded due to missing data on their appendiceal caliber. Nevertheless, inclusion and exclusion criteria were strictly applied and we did not observe significant differences between included and excluded patients in any of the sociodemographic and clinical characteristics evaluated. For a selection bias to occur in this study, most of the excluded patients would have to have a large appendiceal caliber (≥ 6.9 mm), which is unlikely, as the most likely reason for missing is that the appendix cannot be visualized. Second, external validation of the BIDIAP index needs to be assessed before its use is recommended in clinical practice. Until then, it should be noted that, in our sample, has proven to be valid and to have an excellent discriminatory capacity and calibration.

In conclusion, our results showed that the BIDIAP index is an easy-to-use and inexpensive diagnostic tool with excellent diagnostic performance in PAA. Although external validation is necessary, initial results look promising.

### Supplementary Information

Below is the link to the electronic supplementary material.

Supplementary file1 (DOCX 16 KB)

## Data Availability

All data pertaining to this study are available upon justified request through the author in correspondence.

## References

[1] Michelson KA, Reeves SD, Grubenhoff JA, Cruz AT, Chaudhari PP, Dart AH, Finkelstein JA, Bachur RG (2021). Clinical features and preventability of delayed diagnosis of pediatric appendicitis. JAMA Netw Open.

[2] Duman L, Cesur Ö, KumbulDoğuç D, Çelik S, Karaibrahimoğlu A, Savaş MÇ (2020). Diagnostic value of serum pentraxin 3 level in children with acute appendicitis. Ulus Travma Acil Cerrahi Derg.

[3] Kakar M, Delorme M, Broks R, Asare L, Butnere M, Reinis A, Engelis A, Kroica J, Saxena A, Petersons A (2020). Determining acute complicated and uncomplicated appendicitis using serum and urine biomarkers: interleukin-6 and neutrophil gelatinase-associated lipocalin. Pediatr Surg Int.

[4] Yap TL, Fan JD, Ho MF, Choo CSC, Ong LY, Chen Y (2020). Salivary biomarker for acute appendicitis in children: a pilot study. Pediatr Surg Int.

[5] Elmas B, Yildiz T, Yazar H, İlçe Z, Bal C, Özbek B, Yürümez Y (2020). New oxidative stress markers useful in the diagnosis of acute appendicitis in children: thiol/disulfide homeostasis and the asymmetric dimethylarginine level. Pediatr Emerg Care.

[6] Arredondo Montero J, Bardají Pascual C, Bronte Anaut M, López-Andrés N, Antona G, Martín-Calvo N (2022). Diagnostic performance of serum interleukin-6 in pediatric acute appendicitis: a systematic review. World J Pediatr.

[7] Arredondo Montero J, Bardají Pascual C, Antona G, Bronte Anaut M, López-Andrés N, Martín-Calvo N (2022) Diagnostic performance of calprotectin and APPY-1 test in pediatric acute appendicitis: a systematic review and a meta-analysis. Eur J Trauma Emerg Surg. 10.1007/s00068-022-02000-2. (**Epub ahead of print. PMID: 35633377**) 10.1007/s00068-022-02000-2 35633377

[8] Eun S, Ho IG, Bae GE, Kim H, Koo CM, Kim MK, Yoon SH (2021). Neutrophil-to-lymphocyte ratio for the diagnosis of pediatric acute appendicitis: a systematic review and meta-analysis. Eur Rev Med Pharmacol Sci.

[9] Arredondo Montero J, Pérez Riveros BP, Martín-Calvo N (2023) Diagnostic performance of total platelet count, platelet-to-lymphocyte ratio, and monocyte-to-lymphocyte ratio for overall and complicated pediatric acute appendicitis: a systematic review and meta-analysis. Surg Infect (Larchmt). 10.1089/sur.2023.013. (**Epub ahead of print. PMID: 37022749**) 10.1089/sur.2023.013 37022749

[10] Li C, Tian W, Zhao F, Li M, Ye Q, Wei Y, Li T, Xie K (2018). Systemic immune-inflammation index, SII, for prognosis of elderly patients with newly diagnosed tumors. Oncotarget.

[11] Ozdemir A, Baran E, Kutu M, Celik S, Yılmaz M (2023). Could systemic immune inflammation index be a new parameter for diagnosis and disease activity assessment in systemic lupus erythematosus?. Int Urol Nephrol.

[12] AltaliAlhames K, Martín-Sánchez FJ, Ruiz-Artacho P, Ayuso FJ, Trenchs V, Ortiz M, de Zarate M, Navarro C, Fuentes Ferrer M, Fernández C, González Del Castillo J, Bodas A (2021). Diagnostic accuracy of combining C-reactive protein and alvarado Score among 2-to-20-year-old patients with acute appendicitis suspected presenting to emergency departments. Rev Esp Quimioter.

[13] Lu YT, Chen PC, Huang YH, Huang FC (2020). Making a decision between acute appendicitis and acute gastroenteritis. Children (Basel).

[14] Arredondo Montero J, Antona G, Bronte Anaut M, Bardají Pascual C, Ros Briones R, Fernández-Celis A, Rivero Marcotegui A, López-Andrés N, Martín-Calvo N (2022) Diagnostic performance of serum pentraxin-3 in pediatric acute appendicitis: a prospective diagnostic validation study. Pediatr Surg Int 39(1):27. Erratum in: Pediatr Surg Int. 2022 Dec 13; 39(1):49. 10.1007/s00383-022-05289-7. (**PMID: 36454367; PMCID: PMC9713741**) 10.1007/s00383-022-05289-7 PMC9713741 36454367

[15] Arredondo Montero J, Antona G, Rivero Marcotegui A, Bardají Pascual C, Bronte Anaut M, Ros Briones R, Fernández-Celis A, López-Andrés N, Martín-Calvo N (2022) Discriminatory capacity of serum interleukin-6 between complicated and uncomplicated acute appendicitis in children: a prospective validation study. World J Pediatr 18(12):810–817. 10.1007/s12519-022-00598-2. (**Epub 2022 Sep 16. PMID: 36114365; PMCID: PMC9617836**) 10.1007/s12519-022-00598-2 PMC9617836 36114365

[16] Hao TK, Chung NT, Huy HQ, Linh NTM, Xuan NT (2020). Combining ultrasound with a pediatric appendicitis score to distinguish complicated from uncomplicated appendicitis in a pediatric population. Acta Inform Med.

[17] Gudjonsdottir J, Marklund E, Hagander L, Salö M (2021). Clinical prediction scores for pediatric appendicitis. Eur J Pediatr Surg.

[18] Banna GL, Cantale O, Muthuramalingam S, Cave J, Comins C, Cortellini A, Addeo A, Signori A, McKenzie H, Escriu C, Barone G, Chan S, Hicks A, Bainbridge H, Pinato DJ, Ottensmeier C, Gomes F (2022). Efficacy outcomes and prognostic factors from real-world patients with advanced non-small-cell lung cancer treated with first-line chemoimmunotherapy: the Spinnaker retrospective study. Int Immunopharmacol.

[19] Toyoda J, Sahara K, Maithel SK, Abbott DE, Poultsides GA, Wolfgang C, Fields RC, He J, Scoggins C, Idrees K, Shen P, Endo I, Pawlik TM (2022). Prognostic utility of systemic immune-inflammation index after resection of extrahepatic cholangiocarcinoma: results from the U.S. extrahepatic biliary malignancy consortium. Ann Surg Oncol.

[20] Keskin Ç, Dilekçi EN, Üç ZA, Cengiz D, Duran C (2022). Can the systemic immune-inflammation index be used as a novel diagnostic tool in the diagnosis of subacute thyroiditis?. Biomark Med.

[21] Neal JT, Monuteaux MC, Rangel SJ, Barnewolt CE, Bachur RG (2022). Refining sonographic criteria for paediatric appendicitis: combined effects of age-based appendiceal size and secondary findings. Emerg Med J.

[22] Harel S, Mallon M, Langston J, Blutstein R, Kassutto Z, Gaughan J (2022). Factors contributing to non-visualization of the appendix on ultrasound in children with suspected appendicitis. Pediatr Emerg Care.

[23] Unsdorfer KML, An JY, Binkovitz LA (2021). Pediatric appendiceal ultrasound: maintaining accuracy, increasing determinacy and improving clinical outcomes following the introduction of a standardized reporting template. Pediatr Radiol.

